# Emmett on Vesico-Vaginal Fistula

**Published:** 1868-09

**Authors:** 


*Emmett on Vesico-Vaginal Fistula.*—We have great pleasure in announcing the appearance of Emmett's work upon Vesico-Vaginal Fistula, published by Wm. Wood, New York. All those curious in this matter, will take the earliest opportunity to read the work, coming as it does from one so experienced and distinguished in uterine surgery.

